# Liquid biopsies in myeloid malignancies

**DOI:** 10.20517/cdr.2019.88

**Published:** 2019-12-19

**Authors:** Bilal Abdulmawjood, Catarina Roma-Rodrigues, Alexandra R. Fernandes, Pedro V. Baptista

**Affiliations:** UCIBIO, Department of Life Sciences, Faculdade de Ciências e Tecnologia, Universidade NOVA de Lisboa, Campus Caparica, Caparica 2829-516, Portugal.

**Keywords:** Liquid biopsy, circulating tumor cells, circulating tumor DNA, exosomes, microRNAs, leukemia

## Abstract

Hematologic malignancies are the most common type of cancer affecting children and young adults, and encompass diseases, such as leukemia, lymphoma, and myeloma, all of which impact blood associated tissues such as the bone marrow, lymphatic system, and blood cells. Clinical diagnostics of these malignancies relies heavily on the use of bone marrow samples, which is painful, debilitating, and not free from risks for leukemia patients. Liquid biopsies are based on minimally invasive assessment of markers in the blood (and other fluids) and have the potential to improve the efficacy of diagnostic/therapeutic strategies in leukemia patients, providing a useful tool for the real time molecular profiling of patients. The most promising noninvasive biomarkers are circulating tumor cells, circulating tumor DNA, microRNAs, and exosomes. Herein, we discuss the role of assessing these circulating biomarkers for the understanding of tumor progression and metastasis, tumor progression dynamics through treatment and for follow-up.

## Introduction

Hematologic malignancies, characterized as affecting the bone marrow, lymphatic system, and blood cells, are the most common kind of cancer among children and young adults. Leukemia, lymphoma, and myeloma are amongst the most frequent presentations within this group of blood related disorders^[1]^. Throughout the sequential stages of hematopoietic differentiation, there are plenty of occasions for disruptive biomolecular events to occur (e.g., mutations) that will then promote and/or result in dissimilar cancer subtypes and clinical presentations^[2]^.

Leukemia may be defined as a cancer that derives from the bone marrow and affects hematopoiesis^[3]^. This malignancy results from an abnormal proliferation of white blood cells in the bone marrow, which then affects the normal development of cells, and may even spread to other organs such as the spleen, the liver, the meninges, lymph nodes, *etc.*^[4]^. Because of the wide variability of white blood cells in the human body, leukemia differs greatly from other types of cancers and may affect people of all ages, from young children to the elderly. In addition, unlike most cancers, population characteristics, such as age, race, and lifestyle, do not exempt from being at risk. Leukemias account for over 50,000 new cancer cases annually^[5]^. Leukemia is generally divided into two types, depending on how quickly the immature cells (blasts) proliferate: (1) acute leukemia; or (2) chronic leukemia^[6]^. Leukemia can be further grouped considering the cell lineage affected either myeloid or lymphoid. As such, leukemias may be grouped into acute myeloid leukemia (AML), chronic myeloid leukemia (CML), acute lymphoblastic leukemia (ALL), and chronic lymphocytic leukemia (CLL)^[7-11]^. Myelodysplastic syndromes (MDS) constitute a particular cohort of hematopoietic myeloid stem cell conditions that has not been completely recognized as such and, sometimes, lack full characterization. However, MDS show strong features of hematologic malignancies with major impact on the patients’ quality of life^[12,13]^. Herein, we focus on liquid biopsies (LB) within the scope of AML, CML, and MDS.

### Myeloid leukemia

#### AML

Similar to other malignant hematopoietic diseases, AML results from an accumulation of immature myeloid blast cells in the bone marrow that often extends to the circulating blood^[14,15]^. However, AML may also affect other tissues, such as the skin (leukemia cutis). AML is a severe life-threatening disease that may arise in all ages but is more common in the elderly with a median age at diagnosis of approximately 70 years^[16,17]^. AML presentation is highly heterogeneous and individual cases are characterized by a wide variety of cytogenetic and molecular defects. For instance, the French-American-British classification proposes that AML should be classified according to a particular score (M0-M7) according morphological and cytochemical criteria^[18,19]^. For example, about 41% of AML patients have a normal karyotype, but several chromosomal translocations have been described, such as t (15; 17) (q22; q21) in 13% of patients; t (8; 21) (q22; q22) in 7% of reported cases; and inv (16) (p13; q22)/t (16; 16) (p13; q22) in 5% of cases^[18]^. Some of these chromosomal alterations lead to the expression of abnormal fusion proteins with oncogenic potential, or to the inactivation of tumor suppressor genes^[20]^. It should be noted that AML with normal cytogenetics is very heterogeneous in its clinical and molecular features, and its pathogenesis is much less clear than that of AML with chromosomal rearrangements. Two of the most frequent such rearrangements occur in two well-known genes, namely *NPM1* (nucleophosmin protein, NPM1) and *FLT3* (fms-like tyrosine kinase 3 protein, FLT3), whose mutational status support prognostics in the clinical assessment^[21]^.

#### CML

CML is a rare malignant proliferative disease of the hematopoietic system with a worldwide incidence of 10-12 new cases per 100,000 habitants per year^[22]^. The molecular hallmark of CML is the Philadelphia chromosome (Ph) that originates from the reciprocal translocation t (9; 22) (q34; q11), between the breakpoint cluster region (BCR) and the Abelson tyrosine kinase (ABL), which produces a fusion gene *BCR-ABL*^[23]^. This gene yields the BCR-ABL oncoprotein, a constitutively activated tyrosine kinase, capable of recruiting and activating several pathways regulating the transduction of intracellular signals, and ultimately resulting in abnormal cellular adhesion, enhanced proliferation, and inhibition of apoptosis^[24,25]^. Disease progression begins by a chronic phase (3-6 years), an accelerated phase follows characterized by clonal transformation. Ultimately, a short terminal blast phase occurs^[26,27]^. One of the first chemical compounds to be used against CML was Arsenic, which was later replaced by X-ray irradiation of the spleen^[28]^. During the early 1970s, the use of busulfan and hydroxyurea for the treatment of CML, which greatly improved the quality of life during the chronic phase of CML, but without preventing or delaying progression to advanced accelerated phase and blast phase^[29]^. In the late 1970s and early 1980s, bone marrow transplantation (allogeneic hematopoietic stem cell transplantation, allo-HSCT) was introduced that allowed completely eradicating the Ph positive cells in the bone marrow and, thus, provided a cure for CML patients^[30-32]^. In 1983, Interferon alpha (IFNα) was reported to significantly reduce the number of white blood cells and induce a cytogenetic response in some CML patients^[33]^. However, the gold standard in CML treatment began with the discovery of Tyrosine Kinase Inhibitors (TIs), which selectively block the activity of the fusion oncoprotein, radically changing the way clinicians approach this disease. The first TKI used in CML therapy was imatinib, which functions as a competitive inhibitor binding to the ATP pocket in the kinase domain, with response rates of over 95%^[34,35]^. Second and third generations of TKIs, such as dasatinib, nilotinib, bosutinib, and ponatinib, have subsequently been introduced, improving the efficiency of CML chemotherapy^[36]^.

### MDS

With a yearly incidence of 4:100,000 (prevalence 7:100,000), MDS constitute a group of extremely frequent hematological malignancies, characterized by disruption of the bone marrow stroma, with poor differentiation and maturation. The incidence of MDS rises sharply with age, reaching 50:100,000/year above 80 years of age^[37,38]^. However, the median age at onset is around 70 years, with only 10% of MDS diagnosed patients under the age of 50^[37-41]^. There is an important clinical criterion for MDS diagnosis that is a constant cytopenia for more than three months (other conditions that may cause cytopenia must have been excluded), which is usually accompanied by a reduction of blood cell counts. These patients show an increased risk (20%-25%) of developing AML^[41,42]^. For accurate MDS clinical diagnosis, several features are considered: (1) dysplasia in more than 10% of cells within one or more cell lineages; (2) more than 15% of ring sideroblasts; (3) cytogenetic abnormalities; and (4) mutations in the splicing factor SF3B1 in combination with > 5% ring sideroblasts^[43-45]^.

### LB

Biopsies are medical procedures that involve taking a small sample of tissue to be examined and evaluated, and they have been used by clinicians to diagnose and manage diseases for more than 1000 years^[46]^. In cancer patients, biopsies are critical for the full histopathological characterization and staging of the disease, and they may also provide material for the characterization of the genetic profile of the tumor, which is key in enabling the projection of disease progression and the response to therapy^[47]^. Despite current advances in cancer diagnosis and treatment, traditional biopsies require invasive procedures (e.g., surgery), which may have potential complications, sometimes cannot be repeated for sampling, and are limited in use when the patients’ health status has decayed considerably or when the tumor is not accessible^[48]^. The genomic profile of biopsies provides a portrait of the cancer itself and of the considerable genetic heterogeneity of the several subclones that constitute the “tumor”, but they show a tremendous drawback: they are limited to assessing only a snapshot of the tumor rather than the whole dynamic process^[49]^. In fact, it has been widely recognized that the genome context of tumors (and their metastasis) show a dynamic development in time, frequently as a response to the selective pressure of the surrounding (microenvironment) and/or of the challenging chemo- and radiotherapies (acquired resistance) that are used to tackle cancer development and that might trigger the evolution of diverse cell clones^[48]^. For these reasons, in recent years, there has been a strong focus on retrieving a more comprehensive overview of the disease by analyzing tumor material directly in blood^[49]^.

Biopsies of peripheral blood are called LB and represent a novel and more comprehensive approach to obtain robust molecular information from the tumor^[50]^. Apoptotic and necrotic cancer cells release into the blood/lymph circulation plenty of circulating DNA fragments (circulating tumor DNA, ctDNA), together with a multitude of small vesicles, namely exosomes (EXOs), which are small vesicles released by cells that include proteins and nucleic acids^[47-49,51]^. In many situations, LB allow for imaging techniques and/or re-biopsy. Perhaps the biggest benefit of LB is that retrieving the critical sample fluid is much less invasive than their solid counterparts, or even some imaging approaches relying on the use of contrast agents^[52]^. Another advantage of using LB is the possibility of improved early diagnosis. In some advanced staged cancers, ctDNA may be detected in three out of four patients^[53,54]^. A strong case for LBs has been put forward by the European Society for Medical Oncology that has proposed to use LB to re-biopsy for assessing the genomic mutation status of Epidermal Growth Factor (EGFR) in patients^[55,56]^, which is strongly associated to drug response and the development of drug resistance mechanisms for these types of drugs. However, the possibility to assess the molecular evolution of these malignancies makes LBs a valuable tool to screen and identify alterations associated to drug response and, more relevant, profiles correlating to the development of drug resistance.

In fact, it is not the case of LB versus solid biopsies, but rather the complementary information that might be retrieved by using a combination of these approaches. When used together, LB allows for a more comprehensive characterization of tumor heterogeneity, but with a less invasive procedure that decreases the eventual discomfort to the patient. As such, it is possible to retrieve more information on the dynamics of molecular changes of cancer cells^[57]^, which is critical for the assessment of therapeutic follow-up. LB opens up new possibilities for clinical characterization of malignancies for: (1) clinical management of advanced stage cancers; (2) prognosis of response to therapeutics; (3) detection of recurrence; and (4) evaluation of tumor genomic evolution^[47,57]^.

### ctDNA

During their lifespan, cells may release portions of DNA via numerous normal and pathological mechanisms. In normal conditions, cell free DNA (cfDNA) that is release from cells undergoing apoptosis and/or necrosis is rapidly taken up by the immune system, i.e., tissue macrophages^[58,59]^. However, the phagocytosis system may be overwhelmed and, as a result, higher amounts of DNA fragments reach the bloodstream. This DNA degradation frequently occurs during apoptosis, resulting in fragmentation of chromatin into nucleosomes that originate cfDNA fragments of 180-200 base pairs (bp)^[60]^. Living tumor cells also release ctDNA into the bloodstream, which may then mediate further malignant transformation and metastasis^[61]^.

However, differences exist between the length ctDNA and cfDNA fragments. For example, some human cancers present a ctDNA rich fraction encompassing fragments that are circa 20-50 bp shorter than that of cfDNA of healthy donors^[62]^. ctDNA has several molecular characteristics associated with the tumor of origin, such as mutation pattern, copy number variations (CNVs), and methylation level^[63-67]^. ctDNA is predominantly found in plasma and serum, but it has also been recovered from other body fluids, such as breast milk, bone marrow, *etc.*^[68]^. Generally, circulating nucleic acids show half-lives between 15 min and several hours before being removed from circulation by the liver and kidney^[69,70]^.

One of the main concerns when analyzing cfDNA is the possibility of contamination by genomic DNA (e.g., that might be released from circulating blood cells or endothelia). This may be circumvented by using plasma rather than serum as LB for cfDNA analysis^[71]^. There have been several reports putting forward the use of other body fluids, such as urine^[72]^, saliva^[73]^, and cerebrospinal fluid^[74]^, as a substitute for plasma as a source of sizeable amounts of ctDNA. In LB targeting cfDNA, it is recommended to use anticoagulants that may also act as nuclease inhibitors, such as ethylenediaminetetraacetic acid (EDTA). Caution should be paid to the fact that excess EDTA may hamper the efficiency of the downstream enzymatic reactions, such as those used in amplification protocols (e.g., polymerase chain reaction, PCR)^[75]^. The major drawback of using blood as LB is the presence of genomic DNA derived from circulating blood cells that would both affect concentration of cfDNA and introduce bias in terms of sequence analysis^[76]^. In addition, transport and storage conditions affect cfDNA amounts, since non-optimal temperature and mechanical stress might induce cell lysis that release cfDNA into the collected sample^[77]^.

The potential of cfDNA for cancer genetic profiling has been recognized since the end of World War II^[78]^. Some thirty years later, it was already accepted that cancer patients showed elevated levels of cfDNA in peripheral blood. The average cfDNA concentration is almost 400 times higher for cancer patients, when compared to the healthy individuals^[79]^. The highest levels were observed in patients with advanced metastatic disease resistant to chemotherapy and poor prognosis^[79]^. The relevance of cfDNA analysis in hematological malignancies was reinforced by showing that possibility to assess specific mutation status associated to malignancy, such as *NRAS* mutations in MDS and AML^[80]^. In 2013, the first report of cancer detection using cfDNA from a pregnant woman showed the correlation between the non-invasive prenatal testing suggestive of an aneuploidy for chromosomes 13 and 18 (that targets fetal cfDNA in maternal cfDNA) and a small cell carcinoma with aneuploidy in 80% of analyzed cells, including chromosomes 13 and 18^[81,82]^.

Currently, ctDNA analysis is used for both for direct diagnosis and to assess tumor progression, particularly useful to evaluate the molecular response to therapeutics. These analyses can be performed using both manual and automated systems able to extract ctDNA from plasma^[83,84]^. ctDNA has also been used to assess tumor burden and for multiplex analysis of gene mutations, including the customary biomarkers: EGFR, HER-2, BRAF, KRAS, ALK, and KIT^[85]^. Additionally, mounting evidence indicates that ctDNA is extremely useful for the identification of mutations associated to treatment options^[83,86,87]^. In addition, even CNVs, which may be difficult to analyze, have been successfully screened^[85]^. A recent approach has been reported to be used for the diagnostics of eight common cancer types through the assessment of mutations in cfDNA-CancerSEEK^[88]^.

The growing knowledge that a particular tumor may evolve through the expansion of heterogenous genetically distinct subclones originating from sequence alterations has triggered the interest in assessing the frequency, identity, and molecular evolution of these clones. The difficulties in performing these analyses from traditional biopsies has redirected clinicians to use LB, with the possibility to follow the succession of occurring molecular events^[89,90]^. In fact, the evolutionary phylogenetic subclone analysis in non-small cell lung carcinoma (NSCLC) was possible via the utilization of ctDNA collected over 30 weeks^[90]^. From the reported data, it becomes clear that ctDNA may be used for the molecular analysis of subclones of a primary tumor that only exist in small sizes. In addition, the use of ctDNA also allowed characterizing metastatic subclones from the primary tumor that could be used as a specific tumor marker for evaluating response to in NSCLC^[90,91]^.

### Circulating tumor cells

The first description of the ability of cancer cells to travel through the bloodstream and metastasize in distant tissues and organs was reported in the late 19th century^[92]^. During the same period, several studies culminated in the discovery of circulating tumor cells (CTCs) that consisted of tumor cells circulating in the bloodstream whose characteristics strongly resemble those of an existing tumor^[93]^. It is generally accepted that CTCs may be shed from tumor sites, be it the primary tumor or secondary metastatic tissue, directly into circulation. Perhaps it is the relationship between CTCs’ tumorgenicity and their “traveling” capability that has made them a pivotal element in the processes leading to the development of metastases^[94]^. CTCs are nucleated cells originating in epithelia that make their way into the bloodstream and are found circulating in the in low numbers (less than 1 per 100 leukocytes)^[95]^. Depending on the tissue of origin, CTCs may present distinct morphological characteristics that vary according to cancer type and stage^[94]^. CTCs are usually found in clusters of tumor cells or associated to fibroblasts, leukocytes, and other endothelial cells, which increases the odds of colonizing distant tissues and originating metastasis^[96]^. Almost every type of solid tumor has been shown to be capable of shedding CTCs to the bloodstream and, thus, turning these CTCs into valuable biomarkers of disease progression^[97]^. As such, tremendous effort has been directed towards the development of analytical systems able to detect and isolate CTCs from whole blood. The majority of these platforms rely on the presence/absence of specific markers at the cell surface, such as epithelial cell adhesion molecule (EpCAM+), cytokeratin 8 (CK8+), and cytokeratin 19 (CK19+), among others^[98]^. For example, using these biomarkers, it was possible to detect metastatic breast cancer, where a level of 6-7 CTCs per 10 mL of blood correlated to poor survival^[99]^. However, sometimes, CTCs do not exhibit robust EpCAM expression at the surface and, as such, other antibodies targeting distinct cell surface markers may be used for capture and isolation of CTCs^[100]^.

Recent developments have simplified the procedures to retrieve CTCs from blood samples. For example, in 2007, Nagrath *et al*.^[101]^ reported on a platform for capturing CTCs from whole blood via a microﬂuidic device, whose size might allow for point-of-care analysis. In the last years, there have been several attempts to develop integrated microﬂuidic devices for improved recovery of CTCs with higher purity by combining antibody assisted capture with size exclusion protocols^[102]^. For instance, a microﬂuidic chip for CTC isolation from blood-iChip-has been advertised that combines magnetic separation, hydrodynamic sorting, and inertial focusing of CTCs^[103]^.

PCR based protocols have allowed amplification of epithelial cell specific markers after the synthesis of cDNA from total RNA isolated from viable CTCs^[104,105]^. Critical information for improving diagnostics and clinical characterization in cancer patients can be retrieved from the molecular characterization of CTCs, e.g., expression level of gene drivers and single-cell profiling^[106-113]^. Several other biomarkers originating from CTCs have been used for more robust molecular profiling of cancer cells, such as single or multiplexed panels of microRNAs (miRNAs)^[114,115]^.

### miRNA

miRNAs are small (19-24 bp) non-coding RNAs that play a critical role in post-transcriptional modulation of gene expression, whose role in cancer was elucidated at the beginning of the millennium^[116-120]^. The prognostic value of miRNAs in cancer has been highlighted in several studies. In 2005, Calin *et al.*^[120]^ showed for the first time the relevance of miRNAs for diagnostic and the correlation to prognostic. Subsequently, it was demonstrated that miRNAs were transcribed from cancer-related regions associated to DNA amplification, deletion, or translocation^[121]^. Another example is let-7, which, when expressed at lower levels in lung cancer patients, correlates to poor survival^[122]^. These pioneering studies highlighted the role of miRNA in cancer development and the value of assessing their expression for diagnosis and its correlation to prognosis^[123]^.

miRNAs are found in virtually all body fluids^[124,125]^. miRNAs are routinely detected in LB without noticeable changes in molecular and chemical stability^[126-130]^. In fact, miRNAs are extremely resilient to ribonucleases, which may be associated to their ability to bind plasma proteins, such as Argonaute 2 and high-density lipoprotein^[131,132]^. In addition, these miRNAs may be engulfed in secretory vesicles, including circulating exosomes and apoptotic bodies^[128,133-135]^ (see below). Selected plasma/serum circulating miRNAs could be used to discriminate cancer patients (e.g., breast^[136]^, colorectal^[137]^, gastric^[138]^, lung^[139]^, pancreatic^[140]^, and hepatocellular^[141]^) from healthy individuals, making them excellent tools for earlier diagnosis.

In 2007, Caivano *et al.*^[142]^ showed that miRNA155 detected in from body fluids could be used as biomarker for the diagnostics and follow-up of hematologic malignancies through non-invasive biopsy approaches. Their results showed that miRNA155 levels in extracellular vesicles (EVs) were significantly higher in CLL, AML, and Waldenström’s macroglobulinemia patients. They also found that miRNA155 levels in EVs were significantly lower in MDS and multiple myeloma. Moreover, high miRNA155 levels in EVs correlated to higher counts of white blood cell in AML patients.

Several approaches to analyze circulating miRNA levels have since emerged, including RT-qPCR, high-throughput microarrays, and, more recently, deep next generation sequencing (NGS) protocols^[143]^.

### Exosomes

The intrinsic characteristics of exosomes make these vesicles suitable tumor biomarkers for LB. Exosomes are nanosized vesicles composed of a bi-lipidic membrane containing membrane proteins with a lumen composed of cytoplasmic proteins, RNAs, including mRNA and miRNAs, and others, such as cytokines or growth factors. Exosomes are formed in the endosomal pathway of eukaryotic cells and, thus, are highly enriched in proteins, such as tetraspanins, and adhesion molecules, such as integrins and cadherins^[144]^. Exosomes have an important role in cell-cell autocrine, paracrine, and endocrine communications and can be isolated from body fluids, such as plasma, urine, pericardial, pleural effusions, and amniotic fluid^[145,146]^.

In the cancer context, tumor cell derived exosomes have an active role in tumor progression, therapy invasion, and metastasis formation^[147-152]^. As exosomes content depends on the physiological state of the cell, the cargo and abundance of tumor derived exosomes generally reflects the stage of the tumor, rendering diagnostic value in LB for early tumor detection^[153-155]^. Even though exosomes are not being used in clinical practice, the potential of these vesicles for diagnosis is highlighted by the 71 clinical trials studies focused on exosomes of cancer patients (according to www.clinicalTrials.gov accessed on 12 September 2019), accompanied by the development of platforms for tumor derived exosomes detection in complex samples (e.g., surface plasmon resonance for exosome biomarkers detection^[156]^, microfluidic chip for ovarian cancer diagnosis^[157]^, or a platform with superparamagnetic conjunctions and molecular beacons targeting urinary exosomes for prostate cancer diagnosis^[158]^). Nevertheless, several sensitive and specific exosomal biomarkers were already suggested for early diagnosis of ovarian cancer (over-expression of miR-200a, miR-200b, and miR200c in patient’s serum exosomes)^[159-161]^, non-small cell lung cancer (enrichment of Leucine-rich α2-glycoprotein 1 in exosomes of patients urine and MALAT-1 in patient’s blood)^[162-164]^, colon cancer (overexpression of miR-1246 and miR-23a in patient’s serum exosomes)^[165,166]^, or pancreatic ductal adenocarcinoma (presence of glypican-1 in patient’s serum)^[167,168]^. Regarding the metastatic potential of exosomes, it was described that presence of VEGF and PGF in exosomes could induce the initiation of the pre-metastatic niche at distant sites^[169,170]^ and the presence of specific integrins in the membrane of exosomes dictate internalization by a specific cell type, predicting organ of metastasis, e.g., exosomes expressing ITGα_V_β_5_ bind specifically to Kupfer cells, defining liver tropism^[171-173]^.

The interest in the role of exosomes in liquid malignancies has been gaining momentum. As for the solid tumors, exosomes play important roles in progression and therapy resistance of lymphoma and leukemia^[174,175]^. For example, in CML, exosomes are active players in neo-angiogenesis at the bone marrow, possibly through exosome pro-angiogenic miRNAs-miR-92 and miR-126^[176-180]^. The increase density and caliber of microvessels at the bone marrow of CML patients were suggested to be directly correlated with disease progression and poorer prognosis^[181]^.

## LB in myeloid malignancies

Conventional diagnostics of myeloid and/or lymphoid malignancies rely on full blood characterization with precise blood counts, complemented by morphologic examination of the bone marrow. Bone marrow aspirates are usually collected using a fine needle. Other samples include the removal of lymph node. All of these procedures are extremely disturbing for the patient since they are highly invasive^[182]^. Upon therapy, patient follow up requires a peripheral blood assessment every three months for morphological, cytogenetic, or molecular marker evaluation. The mounting evidence supporting the straightforward correlation between blood and biopsies in hematological malignancies has challenged the need for invasive procedures to retrieve information that is accessible via LB^[182]^. The discovery of CTCs and ctDNA has paved the way for non-invasive monitoring techniques. In fact, a peripheral blood sample allows screening CTCs, ctDNA, cfDNA, miRNAs, and vesicles (e.g., exosomes)^[182]^. In the next sections, we focus on the advantages of LB for myeloid malignancy management.

### LB in AML

cfDNA mutational profiling in hematologic malignancies was performed for the first time in patients with AML and MDS^[80]^. Mutations in cfDNA were detected in 60% of patients, whereas these mutations were not present in DNA extracted from the respective paired peripheral blood or bone marrow. This fact reinforced the idea that plasma was an accessible and valuable material for diagnostics and monitoring of AML/MDS^[80]^. More than 20 years later, it was revealed that blood of AML/MDS patients was enriched in tumor DNA^[183]^. Indeed, it is well known that cancer patients and, in particular AML patients, have elevated levels of cfDNA when compared to healthy individuals^[184,185]^. miRNAs seem to be a valuable biomarker in AML, since these small molecules are more abundant in the plasma from AML patients at diagnosis, when compared to healthy individuals. Interestingly, miRNA levels decrease after chemotherapy.

Another interesting element is that AML patients undergoing induction chemotherapy showed a reduction in circulating nucleosomal DNA fragments, but, in patients with complete remission, an initial rise in the cfDNA level was observed between Days 2 and 4 after induction chemotherapy, possibly due to increased apoptosis in response to treatment^[186]^.

A pilot study by Koutova *et al.*^[187]^ in 2015 used plasma for characterization of miRNA signatures in AML patients (at diagnosis and in remission) that could be correlated to evolution following standard chemotherapy and before planned transplantation.

Analyzing cfDNA from peripheral blood and gDNA from bone marrow, it has also been demonstrated that Loss of Heterozygosity and chromosome X inactivation are the most frequent genetic abnormalities found in AML and MDS patients^[183]^. Data retrieved from this type of wide screening studies suggest that analysis of tumor derived cfDNA may replace bone marrow aspirated for screening genomic abnormalities.

In AML patients, cytogenetics is the starting point for full diagnostics characterization. Then, several gene markers have been used for the genetic molecular profiling. Nucleophosmin 1 (*NPM1*), Proto-oncogene C-KIT (*C-KIT*), CCAAT/enhancer binding protein alpha (*CEBPA*), and FMS related tyrosine kinase 3 (*FLT3*) are used for prognosis and direct therapeutic decisions following initial induction chemotherapy^[188]^. *NPM1* is the most commonly mutated gene in AML and is therefore a valuable biomarker of disease^[189,190]^. Quan *et al.*^[189]^ used cfDNA from AML patients to analyze the number of copies and mutations in *NPM1* by qPCR and NGS, respectively. The mutation profile of *NPM1* correlated directly with different subtypes of AML. Cytogenetic and particular molecular profiles found in cfDNA of patients with myeloid disorders seem to be suitable towards molecular profiling that provides the necessary information for optimized prognosis and treatment decisions.

Recently, Nakamura and co-workers demonstrated, for the first time, that non-invasive ctDNA screening was comparable to bone marrow analysis towards identification of patients at high risk for relapse in MDS and AML following allo-HSCT^[191]^. Additionally, this study also demonstrated that serum ctDNA may be used for assessing minimal residual disease in AML and MDS following allo-HSCT and, thus, may alleviate patients from frequent bone marrow punctures.

### LB in MDS

Bone marrow biopsy is the standard protocol for genetic and epigenetic characterization in MDS. However, this is a complicated procedure that is not always easy to perform and/or feasible^[50]^. In these situations, cfDNA would represent a better and less painful alternative to determine the tumor status, allowing for detection of genetic mutations and methylation status of CpG islands in MDS patients by pyrosequencing^[192]^. More recently, detection of specific mutations in cfDNA using PCR and direct sequencing of several loci was also achieved, demonstrating the wide range of application of cfDNA in MDS characterization^[193]^. Yeh *et al.*^[194]^ evaluated cfDNA as a minimally invasive material for the genetic profiling of MDS patients using a panel of 55 genes previously demonstrated to be involved in cancer onset and development. These findings suggest that cfDNA can provide comparable molecular information to bone marrow biopsy, which might be extremely useful when bone marrow biopsy cannot be performed.

### LB in CML

The use of LB has not been thoroughly explored in CML. Nonetheless, exosomes retrieved from blood of CML patients contain amphiregulin, an inducer of EGFR activation in stromal cells located in the bone marrow that might be used as biomarker^[195]^. In turn, stromal cells activated via EGFR increase the secretion of IL-8, which then stimulates the proliferation of CML cells^[195]^. As such, these molecular markers may be used to evaluate the different stages of tumor cell evolution. It must be noted that the majority of studies focusing on CML have been performed using CML cell lineages, i.e., cell models, and validation of data in real patient samples still needs to be performed. Nevertheless, it has been shown that exosomes secreted by CML cells play a critical role in the formation of new vessels, perhaps even have a pivotal responsibility for neo-angiogenesis in the bone marrow of CML patients^[176-180,195,196]^, and thus could be useful as surrogate markers for disease evolution. Several key molecules thought to play a role in angiogenesis have been identified within exosomes derived from CML, such as the pro-angiogenic miRNA92 and miRNA126^[176,179]^. Moreover, it has been demonstrated that exosomes derived from K562 cells are able to induce angiotube formation in human umbilical vein endothelial cells (HUVEC)^[178]^. In addition, exosomes secreted by LAMA84 CML cells induced expression of IL-8 in HUVEC^[180]^. Altogether, these preliminary efforts seem to indicate the value of exosomes retrieved from LB in diagnostics and prognostics in CML.

## The next step in LB in leukemia

The growing interest in LB as a less invasive approach to retrieve valuable molecular information of malignancies, and thus more convenient for patients, has prompted the development of multiple clinical trials to evaluate LB as prognostic and predictive biomarkers (https://clinicaltrials.gov/). Table 1 highlights some of the most relevant clinical trials evaluating the possible use of LB in myeloid malignancies. It should be noted that there are more than 220 currently ongoing clinical trials focusing on LB in all types of cancer, demonstrating the importance of LB in cancer management.

**Table 1:** Clinical trials of LB in myeloid malignancies

Clinical trial	Malignancy	Sample/Biomarker	Summary	Status	Clinical trial ref.
Monitoring ctDNA after chemotherapy in MDS and AML (iCare3)	AML, MDS	Peripheral blood, BM, fingerstick and saliva	ddPCR to quantify peripheral blood plasma MAF in MDS and AML patients before, during and after chemotherapy. Quantification of MAF from fingersticks and saliva samples to determine feasibility of ctDNA for ddPCR	Withdrawn (modified to collect specimens from a bank requiring a separate IRB-approved protocol)	NCT03138395
ctDNA for minimal residual disease in AML after allogeneic hematopoietic stem cell transplant: a multi-center prospective study	AML, MDS	Peripheral blood and BM	Investigate the impact of ctDNA on relapse and prognosis in AML patients after allogeneic hematopoietic stem cell transplant	Recruiting	UMIN 000033003 (UMIN-CTR Clinical Trial)
Circulating miRNAs as disease markers in pediatric cancers	Leukemia, lymphoma and central nervous system	Peripheral blood and cerebrospinal fluid	Evaluate the presence of miRNAs in blood and cerebrospinal fluid of patients with central nervous system tumors, leukemia and lymphoma who are currently on chemotherapy and undergoing blood draws, lumbar punctures and/or reservoir taps for routine clinical care	Unknown	NCT01541800
LB evaluation and repository development at Princess Margaret (LIBERATE)	Lymphoma, leukemia, other solid tumors	Peripheral blood	Develop an institution-wide LB protocol to establish a common process for collecting blood and corresponding archived tumor specimens for future research studies	Recruiting	NCT03702309
Evaluation of ProALL miRNAs in Blood specimen for prediction of ALL relapse risk	B-cell ALL	Peripheral blood	Monitorization capability of miR-451, miR-151-5p, and miR-1290 in blood samples	Recruiting	NCT03000335
Studying tissue and blood samples from patients with AML	AML	BM aspirate, whole blood, buccal cell sample, and BM biopsy	Comparing tissue and blood samples from AML patients to determine the frequency of specific gene markers mutations, aberrant, over-expression and levels of promoter methylation of specific genes in defined cytogenetic subgroups of patients with AML	Unknown	NCT00900224
Identification of biomarkers that are predictive of early ibrutinib treatment failure in high risk TP53 mutated chronic lymphocytic leukemia	CLL	Tumor peripheral blood cells, plasma, tumor genomic DNA and cfDNA	Identification of dynamic molecular markers for early and real time prediction of sustained benefit or no benefit from ibrutinib treatment in CLL harboring TP53 mutations	Recruiting	NCT02827617
Biological characterization of High-Risk CHildhood Cancer in children, adolescents and young adults (MICCHADO)	Leukemia, other solid tumors	Peripheral blood, BM, and cerebrospinal fluid	Identify and characterize the meaningful molecular genetic alterations and immunological features of high-risk childhood, adolescent, and young adult cancers, at diagnosis, during patient treatment and follow-up (time dimension)	Recruiting	NCT03496402
cfDNA in primary cutaneous lymphomas (MATULILA)	Mycosis fungoides, lymphoma, large B-cell, diffuse	Peripheral blood (plasma) and tumor tissue biopsies	Evaluate the possibility of detecting cfDNA in potentially aggressive primary cutaneous lymphomas by dPCR	Unknown	NCT02883517
Clinical application of LB for precise diagnosis and prognosis in lymphoma	Lymphoma	Peripheral blood	To develop LB technology for accurate diagnosis and prognosis judgment of lymphoma by comparing mutations detected by LB and tumor *in situ* biopsy by NGS sequencing of peripheral blood cfDNA and lymphoma tissue DNA	Recruiting	NCT04062877

LB: liquid biopsies; ctDNA: circulating tumor DNA; cfDNA: cell free DNA; MDS: myelodysplastic syndromes; AML: acute myeloid leukemia; ddPCR: droplet digital PCR; MAF: mutant allele frequency; miRNAs: microRNAs; ALL: acute lymphoblastic leukemia; CLL: chronic lymphocytic leukemia; NGS: next generation sequencing; IRB: institutional review board; BM: bone marrow

Some of these clinical trials are directed at the critical points herein discussed. For example, iCARE (NCT03138395) focuses on the need to provide follow-up in patients undergoing chemotherapy. In fact, not achieving minimal residual disease following chemotherapy might be a critical predictor of refractory disease and seems to be associated with the death of 20% of AML and 80% of MDS patients. iCARE addresses an important issue that concerns the use of droplet digital PCR (ddPCR) for quantitative mutant allele frequency (MAF) in plasma for MDS and AML patients before, during, and after chemotherapy treatment. This approach may bring evidence of the important role of non-invasive means of monitorization in phase II and III clinical trials of new therapeutic agents, and cancer progression, providing clinicians with an opportunity to intervene before patients relapse. This clinical trial has been withdrawn based on the need of collecting specimens from a bank that will require approval of an additional protocol for institutional review board, which highlights the need for standardized sample collection protocols for LB.

Clinical trials UMIN000033003 and NCT01541800 address ctDNA and miRNAs in LB, respectively, as a mean of following relapse and prognosis in leukemia patients. For cfDNA, genome-wide copy number evaluation from plasma via shallow or low-coverage sequencing of cfDNA is scalable and cost-effective^[197-201]^ and has recently been shown to be highly prognostic^[201]^.

LB show several advantages for cancer management strategies, such as: (1) minimally invasiveness; (2) better reproduction of the patients’ genetic mutations profile; (3) suitability as companion diagnostics; and (4) sample collection decreases the possibility of contamination with unwanted tissue/cells. Because LB is less invasive, it may be used more recurrently with less negative impact for patients than conventional biopsies^[202]^. Additionally, mounting evidence shows that LB deliver better portrait of the genomic context of the tumor through time, which may be of extreme valuable as companion diagnostics with the possibility to screen for the time dependence evolution of mutations^[203]^. LB further advantages over conventional tissue biopsies include the possibility of becoming a source of fresh tumor-derived material that are free from contamination (e.g., chemical preservatives)^[203]^. The application of LB to follow and assess solid tumors has been clearly demonstrated, whose advantages over tissue biopsies have been thoroughly documented. Newman and co-workers evaluated the performance of LBs as starting material for the screening of genomic mutations and evaluation of disease burden by quantifying cfDNA levels. The molecular data retrieved from these assessments were compared to whole body imaging screens via positron-emission tomography and computed tomography, which showed that lower levels of cfDNA in the plasma correlated with better clinical prognostics^[204]^.

There are also some limitations to the widespread utilization of LB. It should be noted that LB might be less efficacious for detecting rare variants at very low frequency. In addition, LBs may be able to assess tumor heterogeneity and identify which clones dominate at a particular colonization site, but combining them with standard solid biopsies clearly improves the robustness of information. Because different clones at different sites will release ctDNA at distinct rates, LBs may introduce an undesired bias into the quantitative molecular characterization of the tumor evolution. For example, brain metastases usually release little to no ctDNA into circulation^[53]^. This is particularly relevant when considering the assessment of minimal disease and/or resistance of a few clonal cells to therapeutics, where extremely high sensitivity is usually desirable.

In addition, several studies have highlighted the differences in cfDNA levels derived from distinct tumor types and between body fluids used for analysis^[205-207]^. The amount of cfDNA derived from a particular tumor correlates with the cancer stage, degree of vascularization, metastatic potential, and apoptosis rate^[206,208]^. In some cases, tumor-derived cfDNA may be lower than 1% of the total circulating DNA, which may not allow for retrieving the full picture required for effective management of the patient in hand. In addition, identification and characterization of mutations requires extensive validation of processes and protocols so as to ensure clinically relevant data^[205,207]^.

## Conclusion

Early detection of cancer is critical for improving the chances of therapeutic intervention and enhance the overall survival rates. Tumor detection is conﬁrmed by traditional tissue biopsy. Due to their invasiveness, issue biopsies show potential harmful effects on the patient wellbeing with several complications, they are sometimes impossible to repeat over time, and, in some clinical conditions, they are impossible to obtain when the tumor is inaccessible. As such, oncologists have been focusing on cancer-derived material in the blood, which are easier to access and collect with less impact to the patient. LB relying on CTCs, ctDNA, miRNA, and EXOs are at the core of innovative approaches to retrieve relevant information on the molecular aspects of the tumor, offering a range of information that allows functional studies, and with the potential for diagnostics and management of myeloid malignancies, including MDS, AML, and CML. Innovative technology platforms have allowed the combination of standard molecular proﬁling with LB into integrated systems for biomarker capture and analysis. However, CTCs, ctDNA, and EXOs are usually recovered in small concentrations from samples, negatively impacting the analytical output in terms of specificity and sensitivity. Current high-throughput platforms often provide data that are not easy to reproduce across laboratories, highlighting the need for standardization of procedures for collection and processing of LBs in clinical context, together with the analytical validation of the protocols when utilizing LB.

## Declarations

### Copyright

© The Author(s) 2019.
